# A Case of Facial Neuralgia Treated by Extirpation of the Superior Maxillary Nerve

**Published:** 1872-01

**Authors:** B. A. Watson


### ARTICLE VI.

### *A Case of Facial Neuralgia Treated by Extirpation of the Superior Maxillay Nerve.*

BY B. A. WATSON, M. D.

T. De Y., æt. fifty-two years, married, of temperate habits, a rectifier by occupation for the last twenty years; had previously worked at shoemaking. During the early part of his life, while engaged in shoemaking, he suffered severely from indigestion and hepatic derangement, which were usually benefited by laxative doses of the massa de hydrargyro. He has continued to suffer more or less from indigestion, but not nearly so much since he changed his occupation. The neuralgic disease first made its appearance in the spring of 1868. At this time he observed, when he washed his face in cold water, that he had flashes of pain about the region of the right cheek and orbit. The pains now were of short duration, lasting only a few seconds. These pains gradually increased, and he soon began to suffer severely: was unable to eat, drink, converse, or laugh without having a most violent paroxysm, causing him to shriek with anguish. These paroxyms were more severe during the day than the night. After suffering thus acutely for about six months, he found himself partially relieved. (The extraction of teeth, and medicines used, had failed to give any relief.) This temporary relief continued until about four weeks previous to the performance of the operation, when it returned suddenly. The exacerbations assumed a more violent form, marked by excruciating and nearly continuous agony during the day. His sufferings were now much more severe than on any previous occasion. The pain was not now confined to the cheek and orbit, but the lip and nose were involved. The slightest touch upon the surface of the face, a current of air, or a mouthful of water acting on the palate, would throw the patient into a violent paroxysm of agony. The condition of the patient was such that he could not attend to any business; his general health was greatly impaired; he frequently spoke of his life as a burden; feared the loss of his reason. He had tried medicines until he had lost all confidence in their power to relieve his sufferings, and begged for an immediate operation.

I determined to perform Dr. Carnochan's operation, as modified by Prof. Jas. R. Wood. Dr. Carnochan's cases are reported in *American Journal of Medical Science*, vol. xxxv.; Prof. Wood's in *New York Medical Journal*, June, 1871.

I operated June 11th, 1871, with the assistance of Drs. Varick, Mulcahy, Wolfe, McGill, Gardner, Craig, and Gray. The patient was placed in a large arm-chair before an open window, with his head resting upon the shoulder of an assistant, when the ether was administered, and "a semi-lunar incision was made in the right cheek, commencing at the inner, and terminating at the outer angle of the eye. A verticle incision was then made from the centre of the first, extending down to the vermillion border of the lip, but not sufficiently deep to enter the buccal cavity. The flesh was then dissected up, and held in that position by an assistant. The branches of the superior maxillary nerve were dissected out from the soft parts, and an opening made through the anterior wall of the antrum, by means of a trephine, three-fourths of an inch in diameter." I now, at the suggestion of my friend, Dr. Varick, introduced into the infra-orbital foramen a small probe, which I passed backward through the canal; and then, placing the concave surface of a gouge over the probe, and carrying the gouge backward through the infra-orbital plate, the nerve was easily dislodged from its bed in the groove as far back as the posterior wall of the antrum. A smaller trephine was now used for perforating the posterior wall, thus opening into the spheno-maxillary fossa, "exposing the nerve at its exit from the foramen rotundum, and also Meckel's ganglion."

The nerve was then gently drawn forward and divided behind the ganglion, and a portion of nerve two and a half inches in length was removed. The hemorrhage during the operation was very slight. As soon as the oozing had stopped the semi-lunar and the upper portion of the vertical incision being left open for the purpose of drainage.

Two hours after the operation the pulse was eighty-four per minute; skin natural; some nausea, attributable to the anæsthetic; all sensation in the parts supplied by the superior maxillary nerve was completely destroyed.

*June* 12.—Patient has not slept during the night. Pulse eighty-four; skin natural; tongue dry and slightly furred; complains of itching of the alæ nasi, which is not relieved by friction. Ordered potass. bromidi and chloral hydrat. gr. xviii. of each, to be taken at bedtime, and repeated every hour until sleep is produced.

*June* 13.—Took two doses of the medicine in the early part of the evening, and slept until three o'clock this morning; then took another dose, and slept until six o'clock. Feels better this morning; has eaten a good breakfast, which he enjoyed; pulse 84; wound is looking well. Syringed it out with a weak solution of carbolic acid in water. There is no pain, but the itching continues.

*June* 14.—Has taken one dose of medicine during the night; slept well; wound is healing kindly; pulse 84; complains of some neuralgic pains in the left cheek. Removed the pins and applied adhesive plaster.

*June* 15.—Pains in left cheek continue; pulse 90; bowels constipated. Ordered dose of citrate of magnesia.

*June* 16.—Feels better to-day. Bowels have acted. Pulse 84; wound doing well. Ordered tonic doses of sulphate of quinine

*June* 17.—Less swelling about the face than before. Pulse 84.

*June* 19.—Patient out walking to-day; remained out two hours.

*June* 21—Goes out walking daily,and is improving rapidly. Has very little pain of any sort. Pulse 68. Very light discharge of pus from the wound.

*June* 25.—Intends to return to business to-morrow. Wound discharges only a few drops of pus during the day.

He resumed his business the next day without pain or inconvenience.

I desire to add, in connection with the above notes, that cold water dressings were applied to the wound after it was closed, and continued for three days. The wound was carefully syringed out every day for about two weeks. During the first four days there was a slight oozing of blood serum, and after this followed a slight flow of pus. The patient remained in bed only one day. The wound was perfectly healed at the end of six weeks. The general health has continued to improve from the time of the operation to the present date.

The neuralgia which showed itself on the left side of the face has disappeared; it was of short duration and at no time severe, although the patient—a nervous subject—was seriously annoyed by its presence, in anticipations of future sufferings.—*Medical Record.*

---

